# Antagonistic effects of *Plasmodium*-helminth co-infections on malaria pathology in different population groups in Côte d’Ivoire

**DOI:** 10.1371/journal.pntd.0007086

**Published:** 2019-01-10

**Authors:** Eveline Hürlimann, Clarisse A. Houngbedji, Richard B. Yapi, Prisca B. N’Dri, Kigbafori D. Silué, Mamadou Ouattara, Jürg Utzinger, Eliézer K. N’Goran, Giovanna Raso

**Affiliations:** 1 Swiss Tropical and Public Health Institute, Basel, Switzerland; 2 University of Basel, Basel, Switzerland; 3 Centre Suisse de Recherches Scientifiques en Côte d’Ivoire, Abidjan, Côte d’Ivoire; 4 Centre d’Entomologie Médicale et Vétérinaire, Université Alassane Ouattara, Bouaké, Côte d’Ivoire; 5 Unité de Formation et de Recherche Biosciences, Université Félix Hophouët-Boigny, Abidjan, Côte d’Ivoire; Centre hospitalier de Cayenne, FRANCE

## Abstract

**Introduction:**

*Plasmodium* spp. and helminths are co-endemic in many parts of the tropics; hence, co-infection is a common phenomenon. Interactions between *Plasmodium* and helminth infections may alter the host’s immune response and susceptibility and thus impact on morbidity. There is little information on the direction and magnitude of such interactions and results are conflicting. This study aimed at shedding new light on the potential interactions of *Plasmodium* and helminth co-infections on anemia and splenomegaly in different population groups in Côte d’Ivoire.

**Methodology:**

Parasitologic and clinical data were obtained from four cross-sectional community-based studies and a national school-based survey conducted between 2011 and 2013 in Côte d’Ivoire. Six scenarios of co-infection pairs defined as *Plasmodium* infection or high parasitemia, combined with one of three common helminth infections (i.e., *Schistosoma mansoni*, *S*. *haematobium*, and hookworm) served for analysis. Adjusted logistic regression models were built for each scenario and interaction measures on additive scale calculated according to Rothman et al., while an interaction term in the model served as multiplicative scale measure.

**Principal findings:**

All identified significant interactions were of antagonistic nature but varied in magnitude and species combination. In study participants aged 5–18 years from community-based studies, *Plasmodium*-hookworm co-infection showed an antagonistic interaction on additive scale on splenomegaly, while *Plasmodium*-*Schistosoma* co-infection scenarios showed protective effects on multiplicative scale for anemia and splenomegaly in participants aged 5–16 years from a school-based study.

**Conclusions/Significance:**

No exacerbation from co-infection with *Plasmodium* and helminths was observed, neither in participants aged 5–18 years nor in adults from the community-based studies. Future studies should unravel underlying mechanisms of the observed interactions, as this knowledge might help shaping control efforts against these diseases of poverty.

## Author summary

Malaria (due to infection with *Plasmodium* spp.) and parasitic worms (for example soil-transmitted helminths and *Schistosoma* spp.) are common in the tropics. Hence, people are often co-infected, depending on various factors. Interactions between *Plasmodium* and helminth infections may alter immune response and susceptibility of the infected host, and thus impact on morbidity by either making it worse (synergism) or by reducing it (antagonism). Although these co-infections are common, little is known about the direction and magnitude of such interactions. To deepen the understanding of how co-infection could affect morbidity in infected people, we looked at clinical data (i.e., anemia and splenomegaly) in different population groups in Côte d’Ivoire. We did not observe any exacerbation from co-infection with *Plasmodium* and helminths; all identified significant interactions were of antagonistic nature but varied in magnitude and parasite combination. In the light of enhanced control efforts targeting helminthiases, a better understanding about potential effects on susceptibility to malaria in co-endemic areas should be gained and intervention strategies against the two type of diseases be planned in a more integrative manner.

## Introduction

Anemia is multifactorial. However, in low- and middle-income countries it is largely attributable to parasitic diseases, such as malaria and helminth infections [1]. Yet, the etiology of anemia differs between malaria and helminthiases. *Plasmodium* spp., the malaria causing parasite, affects iron and hemoglobin (Hb) levels during different stages of its development within the human host. An increased red blood cell (RBC) death related to *Plasmodium* asexual replication, reduced iron absorption, and decrease in recycling of RBC iron in individuals with inflammation are potential causes of anemia during a malaria episode [2–5]. Helminth infections are less important with regard to systemic inflammation [5]. However, helminth infections may lead to anemia through blood loss from ingestion of blood cells and lacerations on digestive mucosa by hookworms and through *Schistosoma* eggs passing through blood vessels and bladder and intestinal tissues that lead to hematuria or blood in stool [6, 7]. In addition to anemia, splenomegaly is a frequent clinical manifestation of chronic malaria [4] and advanced *Schistosoma mansoni* infections [8].

Helminths and *Plasmodium* spp. are co-endemic in many parts of the tropics. Hence, helminth-*Plasmodium* co-infection is a common phenomenon [9]. In Côte d’Ivoire, for example, a national school-based survey showed a prevalence of 13.5% for *Plasmodium*-soil-transmitted helminth co-infection and 5.6% for *Plasmodium*-*Schistosoma* co-infection [10]. The impact of such co-infections on the pathophysiology is poorly understood. Effects on the host’s health and immune response due to concomitant infection of *Plasmodium* and helminths are complex and depend on various factors (e.g., stage, intensity, force of transmission, sociodemographic characteristics, immune status, and co-morbidities) [9, 11]. To effectively combat *Plasmodium* infection, a timely and strong pro-inflammatory and type 1 immune response is induced in infected humans. Concomitant chronic helminth infection may, however, alter the immune response by down regulating the Th1 pathway toward anti-inflammatory and type 2 responses [11, 12]. This shift may prevent individuals from clinical immunopathology due to inflammation but may, on the other hand, affect malaria parasite clearance [13]. Apart from immunomodulation, other mechanisms such as cohabitation and competition for resources may also influence clinical manifestations in the co-infected host [11]. Potential interactions on pathophysiology, such as anemia and splenomegaly, might be bidirectional, showing antagonism and synergism in co-infected individuals, which might explain conflicting results of recent studies [14]. While several studies conducted in West Africa identified antagonistic effects or reduced odds for malaria-related pathology in *Plasmodium*-helminth co-infected individuals [15–18], synergism with potential exacerbation of anemia and splenomegaly was reported from studies in Kenya and Zimbabwe [19–21].

Interactions on outcomes in individuals with co-morbidities may be determined on additive or multiplicative scales. Most studies looking at co-morbidities do not look at actual interactions but rather compare estimates from regression analysis between morbidity groups and the few studies exploring actual interactions mainly looked at statistical interaction on multiplicative scale introducing a product term into regression analysis and may thus have missed out potential interaction on additive scale. In clinical research, biologic interactions are often assessed within case-control studies through interaction measures on additive scale, as proposed by Rothman et al. [22]. It is further argued that in etiologic epidemiologic research focus should be put on the assessment of biologic interactions expressed as departure form additivity rather than statistical interaction looking at departure from multiplicativity [23]. The measures proposed by Rothman et al. not only identify the direction but also the magnitude of potential departure from additivity (i.e., antagonism or synergism) and are expressed as synergy index (SI), attributable proportion (AP), and relative excess due to interaction (RERI). In practical use a RERI of 0, an AP of 0 and a SI of 1 would indicate no biologic interaction and exact additivity of the individual effects of *Plasmodium* and helminth infection on the disease outcome meaning *Plasmodium* and helminth infections are independent in causing a certain disease outcome. Yet, if the combined effect of *Plasmodium* and helminth infection is larger (or smaller) than the sum of the individual effects there is interaction on an additive scale or at least departure from additivity that would then translate into a RERI>0, AP>0 and SI>1 or a RERI<0, AP<0 and SI<1, respectively, in case of an antagonistic interaction. This approach has found only limited application in infectious disease epidemiology, although methodologies for cross-sectional designs adjusting for other influencing factors by use of logistic regression analysis have been proposed [24]. With regard to helminth co-infections, a former study revealed significant synergistic effects on anemia among *S*. *japonicum*-hookworm co-infected individuals in The Philippines [25]. To date, such interaction measures for helminth and *Plasmodium* co-infections are lacking. The aim of the current study was to shed new light on the potential interactions on additive and multiplicative scale for *Plasmodium* and helminth co-infections on anemia and splenomegaly among two population groups (school-aged children/adolescents and adults) in Côte d’Ivoire.

## Methods

### Ethics statement

The study protocol was approved by the institutional research commissions of the Swiss Tropical and Public Health Institute (Swiss TPH; Basel, Switzerland) and the Centre Suisse de Recherches Scientifiques en Côte d’Ivoire (CSRS; Abidjan, Côte d’Ivoire). Ethical clearance was received from the ethics committees of Basel (EKBB, reference no. 30/11) and Côte d’Ivoire (reference no. 09-2011/MSHP/CNER-P). In addition, permission for conduct of the national school-based survey was obtained from the Ministry of National Education in Côte d’Ivoire. Directors and teachers of the schools, village chiefs of the study communities, and all concerned district, health, and education authorities were informed about the purpose and procedures of the study. Written informed consent was obtained from each participant, while parents/guardians signed on behalf of children aged below 18 years. Children assented orally. Participation was voluntary; hence, individuals could withdraw from the study at any time without further obligation. All participants benefitted from treatment against soil-transmitted helminths with albendazole (single oral dose of 200 mg for children aged 1–2 years and 400 mg for all participants >2 years) given free of charge. Participants diagnosed with *Schistosoma* eggs were treated with praziquantel (40 mg/kg). In schools where the prevalence of *Schistosoma* infection was above 25%, all school children received praziquantel. Individuals with clinical malaria (i.e., tympanic temperature ≥38°C and a positive malaria rapid diagnostic test (RDT)) and/or severe anemia were offered artemisinin-based combination therapy together with paracetamol and an anti-anemic treatment, respectively, if the RDT result was negative.

### Study design and subjects

Four community-based surveys and a national school-based survey were carried out. Surveys followed a cross-sectional design, including parasitologic and clinical examination and questionnaire interviews for sociodemographic information. The four communities surveyed were situated in southern Côte d’Ivoire and had less than 1,000 inhabitants each with main economic and livelihood activity in subsistence farming and cash crop production (i.e., cacao, coffee, and rubber). The village Sahoua (geographic coordinates: 6°19’20” N latitude, 5°10’30” W longitude) and Ancien Carrefour (5°37’40” N, 4°01’15” W) were visited in August and September 2011, as described in more detail elsewhere [17]. Two rural communities, namely La Scierie (6°49’23” N, 3°24’53” W) and Azaguié CNRA (5°36’10” N, 4°00’51” W), were surveyed between May and August 2013. For recruitment, all community members were invited for participation.

For the national school-based study, 94 school localities were visited between November 2011 and February 2012 (i.e., dry season), whereof 93 participated in the survey. In one school the children received deworming shortly before the current study; all analyses were thus based on the results from 92 schools. The localities were selected applying a lattice plus close pairs design [26] and taking into account the ecologic zones, residential area (i.e., a minimum of 20% urban settings), population density, and the presence of a primary school with a minimum of 60 children attending grades 3 to 5 [10, 27, 28]. In each school, a subsample of 60 children was asked to participate in the study.

Côte d’Ivoire is highly endemic for malaria with perennial transmission of *Plasmodium* infections [28, 29], while infections with soil-transmitted helminths (predominantly hookworm) and *Schistosoma* spp. are more focally distributed [10]. In all surveyed communities overall *Plasmodium* prevalence is expected to be high (≥60%) and prevalence of soil-transmitted helminths to be of moderate endemicity (range: 10–50%) [17]. Sahoua has been identified as endemic zone for *S*. *haematobium* infection, while the two communities situated in the Azaguié district are considered to be *S*. *mansoni* endemic [17, 30].

### Field and laboratory procedures

The study procedures were described elsewhere [10, 17, 27, 28]. In brief, every participant was invited to provide a single stool, urine, and finger-prick blood sample. Duplicate Kato-Katz thick smears [31] were prepared from each stool sample and microscopically examined for the presence of eggs of *S*. *mansoni* and soil-transmitted helminths (i.e., *Ascaris lumbricoides*, hookworm, and *Trichuris trichiura*). Urine samples were either processed by a filtration technique for detection of *S*. *haematobium* eggs or, in case of the national school-based study, using reagent strips (Hemastix; Siemens Healthcare Diagnostics GmbH, Eschborn, Germany) to detect microhematuria [32]. From finger-prick blood samples, thin and thick blood films were prepared on microscope slides. Slides were stained with 10% Giemsa and examined under a microscope (x100) with immersion oil by experienced laboratory technicians. Parasitemia (parasites/μl of blood) of the three endemic *Plasmodium* species (i.e., *P*. *falciparum*, *P*. *malariae*, and *P*. *ovale*) were recorded [33]. Additionally, finger-prick blood samples were subjected to an RDT for *P*. *falciparum* (ICT ML01 Malaria Pf kit; ICT Diagnostics, Cape Town, South Africa). Ten percent of the Kato-Katz thick smears, urine filters, and blood films were subjected to quality control and re-examined by a senior laboratory technician. In case of discrepancies of parasite counts, the slides were re-examined by another senior technician and results discussed until agreement was reached. Clinical examination involved palpation of spleen to assess organ enlargement. Hb was measured using a HemoCue device (HemoCue Hb 301 system; Angelholm, Sweden). Anthropometric measurements, including height and weight, were taken from each participant for subsequent analysis of nutritional status. Sociodemographic information of each participant was collected through administration of pre-tested individual rapid appraisal [34] or a household-based questionnaire [35] for school children and community members, respectively.

### Statistical analysis

Data were double-entered and cross-checked using EpiInfo version 3.5.3 (Centers for Disease Control and Prevention; Atlanta, United States of America). Statistical analyses were performed in Stata version 14.0 (Stata Corp.; College Station, United States of America). Data from the community surveys and the national school-based survey were analyzed separately. Within the community data set, we divided the sample into school-aged children/adolescents (5–18 years) and adults (>18 years). All school-aged children/adolescents and adults with complete parasitologic data and Hb measurement were considered for the main analysis and, depending on the outcome, further stratified into participants with anemia and splenomegaly records, respectively.

According to the World Health Organization (WHO), anemia was defined as Hb levels below 11.5 g/dl and 12.0 g/dl in children aged 5–11 years and children aged 12–14 years, respectively, while pregnant women (≥15 years), non-pregnant women (≥15 years), and males (≥15 years) had anemia cut-offs of 11.0, 12.0, and 13.0 g/dl, respectively [36]. Participants having a palpable spleen of grade 1 or higher using a Hackett’s scale [37] were considered to have splenomegaly. Malnutrition in school-aged children/adolescents was defined as a Z-score <-2 in any of the calculated indicators (i.e., height-for-age, body mass index (BMI)-for-age, or weight-for-age) based on STATA macros from WHO child growth standards [38]. In adults, BMI and mid-upper arm circumference (MUAC) were used to define malnutrition according to cut-offs reported by Eddleston et al. [39].

Schools participating in the national school-based survey were classified into different endemicity profiles to address heterogeneity according to the following criteria: (i) *Plasmodium*-endemic: any positive case per school from microscopy or RDT; (ii) *S*. *haematobium*-endemic: any positive case for microhematuria defined as reagent strip positive with an intensity of ≥1+; (iii) *S*. *mansoni*-endemic: any positive case per school detected by microscopy of Kato-Katz thick smears; and (iv) hookworm-endemic: schools with a minimum hookworm infection prevalence of 10% or any case of moderate- or heavy-intensity infection (≥2,000 eggs per gram of stool (EPG)) [7]. Since school-aged children/adolescents from the community-based and the school-based surveys showed very high rates of *Plasmodium* infection (84.4% and 75.0%, respectively), a two-scenario approach was adopted for analysis of interaction measures of the different *Plasmodium*-helminth co-infection categories. The first scenario used “presence or absence” of *Plasmodium* infection, while the second scenario classified participants into “no or lightly-infected” versus participants with high *Plasmodium* parasitemia. Cut-offs for high parasitemia were set at 1,000 and 1,500 parasites/μl of blood for the school-based and the community-based surveys, respectively, taking into account the relationship between *Plasmodium* parasitemia and prevalence of anemia (S1 Fig). Sample size used for interaction analysis thus varied depending on the number of participants with available information of the two clinical outcomes (i.e., anemia and splenomegaly) and belonging to a school endemic to the investigated pair of parasites.

Univariate logistic regression analysis was applied to calculate crude odds ratios (ORs) for anemia and splenomegaly and different explanatories, including sociodemographic, parasitologic (i.e., (co-)infection status), and clinical indicators. For interaction analysis, multivariable logistic regression models were built for each *Plasmodium*-helminth pair, including all major helminth species identified from parasitologic examination, namely *Plasmodium*-*S*. *haematobium*, *Plasmodium*-*S*. *mansoni*, and *Plasmodium*-hookworm co-infection. To adjust for concurrent helminth infections, infection status with soil-transmitted helminths or *Schistosoma*, respectively, was introduced in the multivariable models as a covariate. All models were adjusted for sex, age group, socioeconomic status, and malnutrition that may have an influence on infection status and clinical outcome [34, 40]. Specifically, for schistosomiasis that is caused by two species in Côte d’Ivoire (*S*. *mansoni* and *S*. *haematobium*), we excluded these school-aged children/adolescents from the sample who were infected with *S*. *haematobium* in case interaction between *S*. *mansoni* and *Plasmodium* was assessed and *vice versa* to obtain species-specific estimates. For all *Plasmodium*-helminth pairs, interaction measures on multiplicative scale were assessed through an interaction term in the respective logistic regression model. RERI, AP, and SI and their respective 95% confidence intervals (CIs) and p-values were calculated to assess departure from additivity using the following formulas incorporated in a readily available calculation mask [23]:

$$RERI=e^{{\hat{\beta }}_{1}+{\hat{\beta }}_{2}+{\hat{\beta }}_{3}}\;-\;e^{{\hat{\beta }}_{1}}-e^{{\hat{\beta }}_{2}}+1$$ (1)

$$AP\;=\;\frac{RERI}{{OR}_{A+B+}}$$ (2)

and

$$SI\;=\;\frac{{OR}_{A+B-}-1}{\left({OR}_{A+B-}-1)+({OR}_{A-B+}-1\right)}$$ (3)

where $e^{{\hat{\beta }}_{1}}$ stands for the OR of having condition A (A+B-) relative to the reference category of not having either condition (A-B-), $e^{{\hat{\beta }}_{2}}$ stands for the OR of having condition B (A-B+) relative to the reference category (A-B-) and $e^{{\hat{\beta }}_{3}}$ is the OR of having both conditions (A+B+) relative to the reference (A-B-).

Interaction measures on additive scale are calculated on the assumption that all infection categories (i.e., single and co-infection categories) present an increased risk for an outcome compared to the reference category. In case ORs were lower than 1 for one or several of these risk categories within a model, AP and SI were considered as non-applicable [41]. Significant antagonistic and synergistic interactions on additive scale were defined as SI <1 and SI >1, respectively, with a p-value below 0.05 and a 95% CI not including 1. The same was applied for interactions on multiplicative scale but using the OR of the respective product term.

## Results

### Operational results

**Fig 1:**
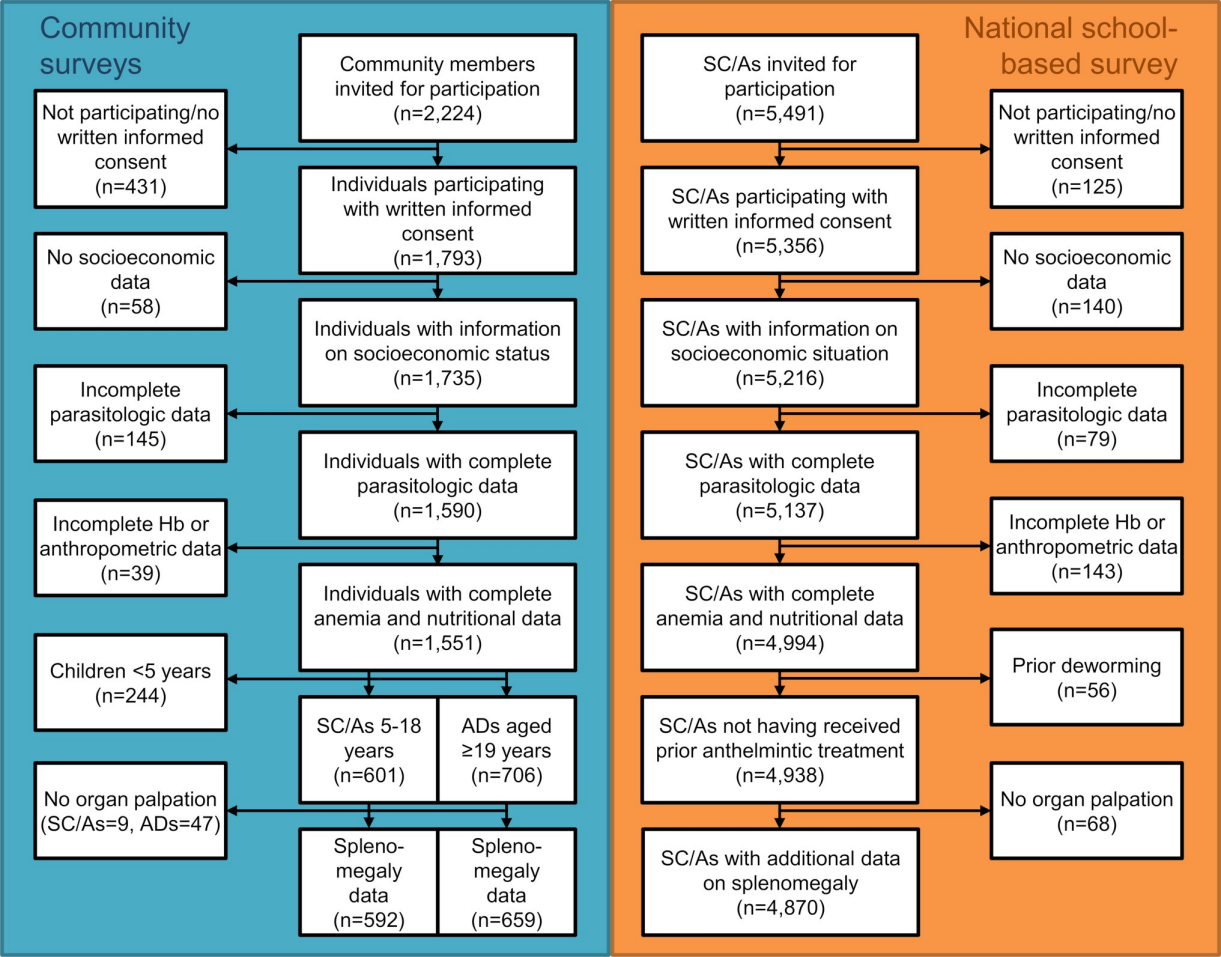
Participation in the community-based and national school-based studies from 2011 to 2013 in Côte d’Ivoire and final school-aged children/adolescents (SC/As) and adult (ADs) samples used for analysis.

Fig 1 depicts the participation in the community- and the school-based studies. From 2,224 invited community members 1,307 (58.8%) participants had written informed consent, complete information on socioeconomic status, parasitologic infection, nutritional indicators, and anemia and were older than 4 years. The community sample for interaction analysis on anemia and splenomegaly consisted of 601 and 592 individuals aged 5–18 years, respectively, while there were 706 and 659 adults, respectively. Among the 5,491 school-aged children/adolescents invited for participation during the national school-based survey 4,938 (89.9%) and 4,870 (88.6%) fulfilled all inclusion criteria for subsequent analysis of interactions on anemia and splenomegaly, respectively.

### Parasite infection and clinical status

Adults, in general, had a lower prevalence of *Plasmodium* and helminth infections than school-aged children/adolescents, with the exception of hookworm that was more often found in older individuals (31.4% in adults compared to 26.0% and 17.2% in school-aged children/adolescents in the community and the school survey, respectively) (Table 1). Anemia among adults (28.3%) from the four communities was comparable to the national school-based survey (28.8%). Splenomegaly showed a considerably lower prevalence in the adult age group (2.7%) compared to school-aged children/adolescents in the school-based (11.7%) and community-based surveys (22.5%). Subsequent interaction analysis for adults was restricted to anemia as a major outcome. From all three investigated population strata (i.e., adults, school-aged children/adolescents from the community-based surveys, and school-aged children/adolescents from the school-based survey), school-aged children/adolescents from the community-based surveys showed the highest rates in terms of infection (prevalence of *Plasmodium*, 84.4%; *Schistosoma*, 42.6%; and soil-transmitted helminths, 28.6%) and clinical outcomes (anemia, 40.4%; splenomegaly, 22.5%). Malnutrition, assessed as a secondary outcome and included in the interaction models as an explanatory factor, was found in 4.7%, 26.0%, and 28.4% of the adults, school-aged children/adolescents from the community-based surveys, and school-aged children/adolescents from the school-based survey, respectively. The national school-based sample revealed a *Plasmodium* infection prevalence of 75.0%. Helminth infections in the school survey were lower compared to the community surveys. While all 92 (100%) schools were classified as *Plasmodium*-endemic, 65 (70.7%), 55 (59.8%), and 36 (39.1%) fulfilled the defined endemicity criteria for *S*. *haematobium*, hookworm, and *S*. *mansoni* infection, respectively.

**Table 1:** Prevalence of parasitic infections and clinical manifestations among adults and school-aged children/adolescents and schools considered endemic for *Plasmodium* and helminth infection, as assessed between 2011 and 2013 in Côte d’Ivoire.

Community-based studies	Adults (n = 706)		School-aged children/adolescents (n = 601)	
	N positive	Prevalence %	N positive	Prevalence %
* Plasmodium* spp.	322	45.6	507	84.4
* Plasmodium*_1500+_	29	4.1	157	26.1
* Schistosoma haematobium*	34	4.8	103	17.1
* Schistosoma mansoni*	147	20.8	157	26.1
* Schistosoma* spp.	178	25.2	256	42.6
Hookworm	222	31.4	156	26.0
* Ascaris lumbricoides*	18	2.6	27	4.5
* Trichuris trichiura*	7	1.0	4	0.7
Soil-transmitted helminths	236	33.4	172	28.6
Anemia	200	28.3	243	40.4
Splenomegaly^a^	18	2.7	133	22.5
Malnutrition	33	4.7	156	26.0
**National school-based study**	**School-aged children/adolescents (n = 4,938)**			
Overall prevalence of parasitic infections/clinical signs	**N positive**	**Prevalence %**		
*Plasmodium* spp.	3,701	75.0		
*Plasmodium*_1000+_	792	16.0		
*Schistosoma haematobium* (microhematuria≥1+)	279	5.7		
*Schistosoma mansoni*	182	3.7		
*Schistosoma* spp.	447	9.1		
Hookworm	851	17.2		
*Ascaris lumbricoides*	90	1.8		
*Trichuris trichiura*	61	1.2		
Soil-transmitted helminths	943	19.1		
Anemia	1,421	28.8		
Splenomegaly^b^	569	11.7		
Malnutrition	1,401	28.4		
	**Schools (n = 92)**			
Endemicity at school level^c^	**N endemic**	**Proportion %**		
*Plasmodium* spp.	92	100.0		
*Schistosoma haematobium*	65	70.7		
*Schistosoma mansoni*	36	39.1		
Hookworm	55	59.8		

^a^Based on 659 adults and 592 school-aged children/adolescents

^b^Based on n = 4,870

^c^Endemicity criteria: *Plasmodium* = any positive case per school from microscopy or RDT; *S*. *haematobium* = any positive case for microhematuria (≥1+) per school; *S*. *mansoni* = any positive case per school detected by microscopy of Kato-Katz thick smears; and hookworm = any school with a prevalence of ≥10% or any case of moderate- or heavy intensity infection (≥2,000 EPG)

### Sociodemographic, parasitologic, and nutritional determinants of clinical morbidity among school-aged children/adolescents and adults

Univariate relationship analysis between clinical morbidity and sociodemographic, parasitologic, and nutritional factors revealed that the contribution from *Plasmodium* and helminth infections on anemia in adults was low (Table 2). Other factors such as older age (>60 years; OR 1.71, 95% CI 1.01, 2.91) and sex (male sex; OR 0.63, 95% CI 0.45, 0.88) ranked much higher among the investigated determinants of anemia. Adult individuals infected with *Schistosoma* (OR 0.67, 95% CI 0.45, 0.99) or co-infected with *Plasmodium* and *S*. *mansoni* (OR 0.49, 95% CI 0.25, 0.95) showed lower crude ORs for anemia. Within school-aged children/adolescents from the community- and school-based surveys, sociodemographic determinants were much more important for school-aged children/adolescents from the school-based sample, which captures a wider range of living conditions, including urban and rural environments and areas with a great variety in ethnic groups and cultures. School children from wealthier households showed lower crude ORs for both anemia (OR 0.74, 95% CI 0.61, 0.91) and splenomegaly (OR 0.50, 95% CI 0.37, 0.67). Anemia was positively associated with male sex (OR 1.32, 95% CI 1.16, 1.49) in the school-based sample. With regard to single parasite infections, *Plasmodium* infection and high parasitemia showed the highest ORs for anemia, and hence, was the main contributor to splenomegaly with 2-fold and 3-fold higher ORs among school-aged children/adolescents from the school- and community-based samples, respectively. In school-aged children/adolescents from the community-based surveys, no significant positive association between clinical morbidity and helminth infections could be shown from univariate analysis. On the contrary, infection with *Schistosoma* and hookworm both showed lower ORs for splenomegaly. In school-aged children/adolescents from the school-based survey, however, infection with *S*. *haematobium* (OR 1.62, 95% CI 1.26, 2.07), *S*. *mansoni* (OR 1.73, 95% CI 1.28, 2.35), and *A*. *lumbricoides* (OR 1.83, 95% CI 1.20, 2.79) showed higher odds for anemia. We found higher odds of anemia and splenomegaly for *Plasmodium*-helminth (*S*. *haematobium*, *S*. *mansoni*, and hookworm) co-infection compared to school children not infected with *Plasmodium* and the respective helminth species. In school-aged children/adolescents enrolled in the community surveys, only the *Plasmodium*-*S*. *haematobium* co-infection showed a significant positive relationship with splenomegaly. Malnutrition showed a positive association with clinical morbidity among school-aged children/adolescents.

**Table 2:** Crude odds ratios (ORs) of sociodemographic, parasitologic, and nutritional factors for anemia and splenomegaly in school-aged children/adolescents (SC/As) and adults (ADs) examined between 2011 and 2013 in Côte d’Ivoire.

Explanatory variable	Community-based studies			National school-based study	
	Anemia in SC/As (n = 601)	Splenomegaly in SC/As (n = 592)	Anemia in ADs (n = 706)	Anemia (n = 4,938)	Splenomegaly (n = 4,870)
	OR (95% CI)	OR (95% CI)	OR (95% CI)	OR (95% CI)	OR (95% CI)
Sociodemography					
Sex (male)	1.10 (0.79, 1.52)	1.49 (1.01, 2.19)*	0.63 (0.45, 0.88)*	1.32 (1.16, 1.49)*	1.15 (0.96, 1.37)
Age group (≥10 years)	0.75 (0.54, 1.04)	0.23 (0.15, 0.37)*	N/A	0.97 (0.86, 1.10)	0.84 (0.71, 1.00)
Age group (≥60 years)	N/A	N/A	1.71 (1.01, 2.91)*	N/A	N/A
Socioeconomic status (very poor)	0.75 (0.46, 1.24)	0.90 (0.49, 1.68)	1.11 (0.68, 1.83)	0.88 (0.73, 1.07)	0.87 (0.67, 1.12)
Socioeconomic status (poor)	0.57 (0.34, 0.96)*	1.07 (0.58, 1.99)	0.88 (0.53, 1.47)	0.96 (0.80, 1.17)	0.78 (0.60, 1.02)
Socioeconomic status (less poor)	0.83 (0.50, 1.37)	1.05 (0.57, 1.95)	1.15 (0.71, 1.87)	0.87 (0.72, 1.04)	0.75 (0.58, 0.97)*
Socioeconomic status (least poor)	0.68 (0.41, 1.14)	1.51 (0.83, 2.74)	0.95 (0.56, 1.60)	0.74 (0.61, 0.91)*	0.50 (0.37, 0.67)*
Residential zone (urban)	N/A	N/A	N/A	0.88 (0.76, 1.02)	0.66 (0.52, 0.83)*
Parasitic infections					
*Plasmodium*	1.17 (0.74, 1.85)	3.50 (1.65, 7.43)*	0.99 (0.72, 1.38)	1.75 (1.50, 2.04)*	2.15 (1.68, 2.74)*
*Plasmodium* (high parasitemia)^a^	1.79 (1.24, 2.59)*	1.86 (1.23, 2.81)*	1.15 (0.51, 2.56)	1.53 (1.33, 1.76)*	1.61 (1.33, 1.95)*
*Schistosoma haematobium*	1.42 (0.93, 2.18)	0.88 (0.52, 1.48)	0.77 (0.34, 1.73)	1.62 (1.26, 2.07)*	1.30 (0.92, 1.84)
*Schistosoma mansoni*	0.73 (0.50, 1.07)	0.64 (0.40, 1.03)	0.68 (0.44, 1.04)	1.73 (1.28, 2.35)*	1.06 (0.67, 1.67)
*Schistosoma* spp.	0.96 (0.69, 1.33)	0.63 (0.42, 0.95)*	0.67 (0.45, 0.99)*	1.69 (1.39, 2.07)*	1.24 (0.93, 1.65)
Hookworm	1.33 (0.92, 1.92)	0.56 (0.35, 0.92)*	0.94 (0.66, 1.34)	1.11 (0.94, 1.30)	0.86 (0.68, 1.10)
*Ascaris lumbricoides*	0.86 (0.39, 1.91)	1.22 (0.50, 2.95)	0.50 (0.14, 1.74)	1.83 (1.20, 2.79)*	1.40 (0.79, 2.50)
*Trichuris trichiura*	N/A	3.49 (0.49, 25.00)	1.01 (0.20, 5.26)	0.96 (0.54, 1.68)	0.82 (0.35, 1.92)
Soil-transmitted helminths	1.12 (0.78, 1.61)	0.60 (0.38, 0.95)*	0.91 (0.64, 1.30)	1.15 (0.98, 1.34)	0.91 (0.72, 1.14)
Co-infections					
*Plasmodium*-*S*. *haematobium*	1.79 (0.93, 3.45)	3.32 (1.24, 8.86)*	0.68 (0.19, 2.49)	2.76 (2.02, 3.77)*	2.75 (1.76, 4.30)*
*Plasmodium*-*S*. *mansoni*	0.91 (0.50, 1.66)	2.15 (0.88, 5.23)	0.49 (0.25, 0.95)*	2.88 (2.02, 4.12)*	1.84 (1.04, 3.26)*
*Plasmodium*-hookworm	1.52 (0.85, 2.74)	2.51 (0.90, 6.98)	0.89 (0.54, 1.47)	1.81 (1.46, 2.33)*	1.75 (1.26, 2.43)*
Clinical indicators					
Malnutrition (z-score <-2)	1.76 (1.22, 2.54)*	2.18 (1.44, 3.29)*	1.69 (0.82, 3.46)	1.43 (1.25, 1.64)*	1.07 (0.88, 1.29)

Reference categories used in logistic regression analysis: Sex = female, age group (SC/As) = <10 years, age group (ADs) = 19–59 years, socioeconomic status = most poor, parasitic infection status = not infected, co-infections = not infected with either of the two species, malnutrition = z-score ≥-2.

^a^Definition of high parasitemia: (i) community surveys: parasitemia ≥1,500 parasites/μl of blood; (ii) national school survey: parasitemia ≥1,000 parasites/μl of blood; reference group = <1,500 parasites/μl of blood and <1,000 parasites/μl, respectively.

*Statistically significant with p<0.05.

N/A = not applicable for this sample.

**Fig 2:**
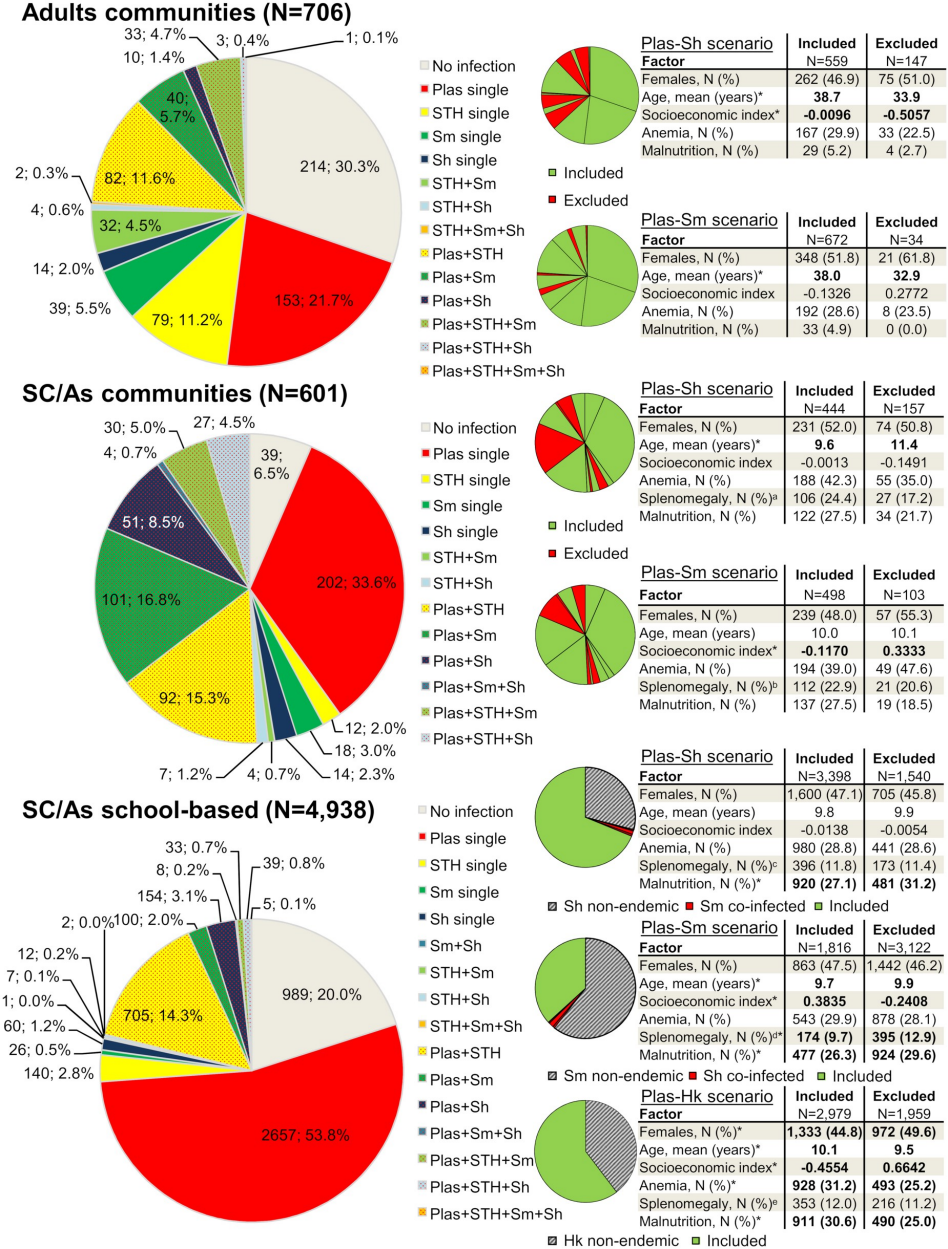
Multiple species infection profiles in adults, school-aged children/adolescents (SC/As) from communities and SC/As from the national school-based survey, subsamples drawn for each *Plasmodium*-helminth scenario and potential differences between included and excluded individuals for interaction analysis. Parasite species abbreviations: *Plasmodium* (Plas), *S*. *mansoni* (Sm), *S*. *haematobium* (Sh), soil-transmitted helminth (STH) and hookworm (Hk). *Statistically significant with a p-value<0.05 according to Χ^2^-test or t-test as appropriate. ^a^Based on a total of 592 whereof 435 were included and 157 excluded. ^b^Based on a total of 592 whereof 490 were included and 102 excluded. ^c^Based on a total of 4,870 whereof 3,347 were included and 1,523 excluded. ^d^Based on a total of 4,870 whereof 1,801 were included and 3,069 excluded. ^e^Based on a total of 4,870 whereof 2,933 were included and 1,937 excluded.

Fig 2 depicts in detail the different multiple species infections observed in the three population cohorts (i.e., adults from communities, school-aged children/adolescents from communities, and school-aged children/adolescents from the school-based survey) and how samples were drawn within to serve for interaction analysis of the different co-infection scenarios. Highest proportions of *Plasmodium*-helminth co-infections were observed in school-aged children/adolescents from the community-based surveys. Excluded individuals in the communities showed differences in mean age and socioeconomic status but were similar with regard to the outcome variables and other influencing factors. Most individuals excluded from the heterogeneous national school-based repository were eliminated during harmonization for specific helminth species endemicity, while only a small proportion (<3%) was dropped for being co-infected with the second *Schistosoma* species. While for the *Plasmodium*-*S*. *mansoni* scenario excluded individuals were of older age, lower socioeconomic status and more affected by co-morbidities (here: splenomegaly and malnutrition) the opposite was observed for the *Plasmodium*-hookworm scenario. Individuals from hookworm non-endemic areas had higher proportions of females, were younger, wealthier, and less affected by anemia and malnutrition.

### Interaction measures on anemia

In all three *Plasmodium*-helminth co-infection models of the adult sample, at least one co-infection had a lower odds (OR <1) compared to the reference category (S1 Table). Consequently, measures of interaction on additive scale were not reliable and their calculation not applicable. We identified a significant antagonistic effect on anemia on multiplicative scale in adult participants co-infected with *Plasmodium* and *S*. *mansoni* (OR 0.39, 95% CI 0.16, 0.95).

Similarly to the findings from univariate regression analysis, single and co-infection categories of *Plasmodium* and *Schistosoma* showed significantly higher ORs for anemia in the multivariable models compared to non-infected school-aged children/adolescents from the school-based survey, while no such relationships could be shown for hookworm infection (Table 3). ORs of the co-infection categories remained slightly below the expected sum of the ORs for the single infection categories, thus showing a tendency for antagonism (SI <1). Indeed, for the scenario of high *Plasmodium* parasitemia and concomitant *S*. *haematobium* infection (OR 0.26, 95% CI 0.13, 0.52), we identified a significant antagonistic effect on multiplicative scale for anemia among school-aged children/adolescents from the school-based sample. This finding was underlined by a negative RERI of -1.86 (95% CI -2.75, -0.96). No significant antagonism or synergism on anemia on additive scale (indicated by SI and AP) for *Plasmodium*-helminth co-infection was identified.

**Table 3:** Interaction measures on anemia and splenomegaly for *Plasmodium*-helminth co-infection among school-aged children/adolescents from a national school-based survey conducted between November 2011 and February 2012 in Côte d’Ivoire.

**Anemia**				
**co-infection categories**	**N anemic/non-anemic**	**OR (95% CI)**	**Interaction measures**^d^	
*Plasmodium-S*. *haematobium* (n = 3,398)^a^			RERI (95% CI) =	-0.14 (-1.54, 1.24)
P negative/Sh negative	142/619	1.00	AP (95% CI) =	-0.05 (-0.53, 0.43)
P positive/Sh negative	736/1,636	1.91 (1.55, 2.35)*	SI (95% CI) =	0.93 (0.47, 1.85)
P negative/Sh positive	24/48	2.17 (1.28, 3.68)*	Product term P x Sh:	
P positive/Sh positive	78/115	2.94 (2.08, 4.15)*	OR (95% CI) =	0.71 (0.39, 1.30)
*Plasmodium*_1000+_*-S*. *haematobium* (n = 3,398)^a^			RERI (95% CI) =	-1.86 (-2.75, -0.96)*
P <1,000/Sh negative	614/1,784	1.00	AP (95% CI) =	N/A
P ≥1,000/Sh negative	264/471	1.59 (1.33, 1.91)*	SI (95% CI) =	N/A
P <1,000/Sh positive	88/118	2.18 (1.63, 2.92)*	Product term P_1000+_ x Sh:	
P ≥1,000/Sh positive	14/45	0.92 (0.50, 1.68)	OR (95% CI) =	0.26 (0.13, 0.52)*
*Plasmodium*-*S*. *mansoni* (n = 1,816)^b^			RERI (95% CI) =	-0.33 (-2.17, 1.51)
P negative/Sm negative	86/369	1.00	AP (95% CI) =	-0.12 (-0.82, 0.58)
P positive/Sm negative	390/805	1.96 (1.49, 2.57)*	SI (95% CI) =	0.84 (0.33, 2.16)
P negative/Sm positive	11/22	2.08 (0.95, 4.54)	Product term P x Sm:	
P positive/Sm positive	56/77	2.70 (1.76, 4.16)*	OR (95% CI) =	0.66 (0.28, 1.57)
*Plasmodium*_1000+_-*S*. *mansoni* (n = 1,816)^b^			RERI (95% CI) =	-0.38 (-1.89, 1.13)
P <1,000/Sm negative	315/942	1.00	AP (95% CI) =	-0.17 (-0.93, 0.59)
P ≥1,000/Sm negative	161/232	1.96 (1.53, 2.49)*	SI (95% CI) =	0.77 (0.24, 2.42)
P <1,000/Sm positive	45/74	1.66 (1.11, 2.48)*	Product term P_1000+_ x Sm:	
P ≥1,000/Sm positive	22/25	2.24 (1.23, 4.08)*	OR (95% CI) =	0.69 (0.33, 1.44)
*Plasmodium*-hookworm (n = 2,979)^c^			RERI (95% CI) =	-0.22 (-0.79, 0.36)
P negative/Hk negative	126/368	1.00	AP (95% CI) =	-0.17 (-0.61, 0.27)
P positive/Hk negative	568/1,172	1.40 (1.11, 1.76)*	SI (95% CI) =	0.58 (0.18, 1.89)
P negative/Hk positive	35/84	1.12 (0.71, 1.76)	Product term P x Hk:	
P positive/Hk positive	199/427	1.30 (0.99, 1.71)	OR (95% CI) =	0.83 (0.51, 1.36)
*Plasmodium*_1000+_-hookworm (n = 2,979)^c^			RERI (95% CI) =	-0.25 (-0.73, 0.24)
P <1,000/Hk negative	484/1,169	1.00	AP (95% CI) =	-0.22 (-0.70, 0.27)
P ≥1,000/Hk negative	210/371	1.34 (1.10, 1.65)*	SI (95% CI) =	0.33 (0.02, 7.34)
P <1,000/Hk positive	178/396	1.02 (0.83, 1.27)	Product term P_1000+_ x Hk:	
P ≥1,000/Hk positive	56/115	1.12 (0.80, 1.59)	OR (95% CI) =	0.82 (0.54, 1.24)
**Splenomegaly**				
**co-infection categories**	**N enlarged/normal spleen**	**OR (95% CI)**	**Interaction measures**^d^	
*Plasmodium-S*. *haematobium* (n = 3,347)^a^			RERI (95% CI) =	-0.15 (-1.89, 1.59)
P negative/Sh negative	47/704	1.00	AP (95% CI) =	-0.06 (-0.71, 0.60)
P positive/Sh negative	310/2,025	2.08 (1.51, 2.87)*	SI (95% CI) =	0.92 (0.35, 2.39)
P negative/Sh positive	8/64	1.78 (0.80, 3.96)	Product term P x Sh:	
P positive/Sh positive	31/158	2.71 (1.66, 4.43)*	OR (95% CI) =	0.73 (0.30, 1.79)
*Plasmodium*_1000+_*-S*. *haematobium* (n = 3,347)^a^			RERI (95% CI) =	-1.52 (-2.73, -0.30)*
P <1,000/Sh negative	232/2,127	1.00	AP (95% CI) =	-1.45 (-3.66, 0.76)
P ≥1,000/Sh negative	125/602	1.81 (1.42, 2.29)*	SI (95% CI) =	0.03 (0.0, 9329144.0)
P <1,000/Sh positive	33/171	1.76 (1.18, 2.62)*	Product term _P1000+_ x Sh:	
P ≥1,000/Sh positive	6/51	1.05 (0.44, 2.48)	OR (95% CI) =	0.33 (0.13, 0.86)*
*Plasmodium*-*S*. *mansoni* (n = 1,801)^b^			RERI (95% CI) =	-2.25 (-5.77, 1.28)
P negative/Sm negative	25/425	1.00	AP (95% CI) =	-1.11 (-3.02, 0.80)
P positive/Sm negative	128/1,059	1.87 (1.19, 2.94)*	SI (95% CI) =	0.31 (0.07, 1.31)
P negative/Sm positive	6/27	3.39 (1.25, 9.19)*	Product term P x Sm:	
P positive/Sm positive	15/116	2.02 (1.01, 4.03)*	OR (95% CI) =	0.32 (0.10, 0.99)*
*Plasmodium*_1000+_-*S*. *mansoni* (n = 1,801)^b^			RERI (95% CI) =	-0.18 (-1.66, 1.29)
P <1,000/Sm negative	111/1,135	1.00	AP (95% CI) =	-0.14 (-1.31, 1.04)
P ≥1,000/Sm negative	42/349	1.12 (0.77, 1.65)	SI (95% CI) =	0.66 (0.02, 24.38)
P <1,000/Sm positive	15/102	1.42 (0.79, 2.57)	Product term P_1000+_ x Sm:	
P ≥1,000/Sm positive	6/41	1.36 (0.56, 3.34)	OR (95% CI) =	0.85 (0.29, 2.54)
*Plasmodium*-hookworm (n = 2,933)^c^			RERI (95% CI) =	0.18 (-0.51, 0.86)
P negative/Hk negative	41/447	1.00	AP (95% CI) =	N/A
P positive/Hk negative	233/1,482	1.68 (1.18, 2.39)*	SI (95% CI) =	N/A
P negative/Hk positive	6/112	0.57 (0.24, 1.39)	Product term P x Hk:	
P positive/Hk positive	73/539	1.43 (0.94, 2.16)	OR (95% CI) =	1.49 (0.59, 3.75)
*Plasmodium*_1000+_-hookworm (n = 2,933)^c^			RERI (95% CI) =	0.35 (-0.41, 1.12)
P <1,000/Hk negative	182/1,447	1.00	AP (95% CI) =	N/A
P ≥1,000/Hk negative	92/482	1.48 (1.13, 1.95)*	SI (95% CI) =	N/A
P <1,000/Hk positive	50/512	0.75 (0.53, 1.05)	Product term P_1000+_ x Hk:	
P ≥1,000/Hk positive	29/139	1.59 (1.02, 2.46)*	OR (95% CI) =	1.43 (0.81, 2.52)

^a^*S*. *mansoni-*positive individuals excluded from model; ORs are adjusted for sex, age group, socioeconomic status, residential zone, soil-transmitted helminth infection, and malnutrition

^b^*S*. *haematobium-*positive individuals excluded from model; ORs are adjusted for sex, age group, socioeconomic status, residential zone, soil-transmitted helminth infection, and malnutrition

^c^ORs are adjusted for sex, age group, socioeconomic status, residential zone, *Schistosoma* infection, and malnutrition

^d^Interaction measures on additive scale: relative excess risk due to interaction (RERI), attributable proportion (AP), and synergy index (SI). Interaction measure on multiplicative scale assessed by product term introduced in the multivariable logistic model

*Statistically significant with p <0.05

Parasite species abbreviations: *Plasmodium* (P), high *Plasmodium* parasitemia with ≥1,000 parasites/μl of blood (P_1000+_), *S*. *mansoni* (Sm), *S*. *haematobium* (Sh), and hookworm (Hk).

N/A = not applicable due to OR <1 for one or more co-infection categories

Interaction analysis in school-aged children/adolescents from the communities revealed no significant interactions, neither on additive nor on multiplicative scale, for anemia as an outcome using six different co-infection scenarios (Table 4). Of note, certain co-infection categories showed significantly higher ORs for anemia compared to the corresponding reference categories such as school-aged children/adolescents positive for *Plasmodium* and *S*. *haematobium* (OR 2.39, 95% CI 1.08, 5.32) and school-aged children/adolescents with high *Plasmodium* parasitemia and concomitant hookworm infection (OR 2.35, 95% CI 1.19, 4.62).

**Table 4:** Interaction measures on anemia and splenomegaly for *Plasmodium*-helminth co-infection among school-aged children/adolescents from four communities examined between 2011 and 2013 in southern Côte d’Ivoire.

**Anemia co-infection categories**	**N anemic/non-anemic**	**OR (95% CI)**	**Interaction measures**^d^	
*Plasmodium-S*. *haematobium* (n = 444)^a^			RERI (95% CI) =	-2.09 (-6.09, 1.90)
P negative/Sh negative	14/37	1.00	AP (95% CI) =	-0.88 (-2.53, 0.78)
P positive/Sh negative	127/167	1.76 (0.88, 3.54)	SI (95% CI) =	0.40 (0.11, 1.40)
P negative/Sh positive	9/12	3.72 (1.26, 11.02)*	Product term P x Sh:	
P positive/Sh positive	35/43	2.39 (1.08, 5.32)*	OR (95% CI) =	0.36 (0.11, 1.21)
*Plasmodium*_1500+_*-S*. *haematobium* (n = 444)^a^			RERI (95% CI) =	-0.44 (-2.47, 1.60)
P <1,500/Sh negative	94/159	1.00	AP (95% CI) =	-0.23 (-1.45, 1.00)
P ≥1,500/Sh negative	45/47	1.57 (0.95, 2.60)	SI (95% CI) =	0.68 (0.09, 5.05)
P <1,500/Sh positive	37/42	1.79 (1.05, 3.06)*	Product term P_1500+_ x Sh:	
P ≥1,500/Sh positive	10/10	1.93 (0.76, 4.89)	OR (95% CI) =	0.68 (0.23, 2.08)
*Plasmodium*-*S*. *mansoni* (n = 498)^b^			RERI (95% CI) =	-1.62 (-4.16, 0.93)
P negative/Sm negative	14/37	1.00	AP (95% CI) =	-1.19 (-2.85, 0.47)
P positive/Sm negative	127/167	1.87 (0.94, 3.75)	SI (95% CI) =	0.18 (0.02, 1.40)
P negative/Sm positive	9/13	2.11 (0.72, 6.18)	Product term P x Sm:	
P positive/Sm positive	44/87	1.36 (0.65, 2.86)	OR (95% CI) =	0.35 (0.11, 1.09)
*Plasmodium*_1500+_-*S*. *mansoni* (n = 498)^b^			RERI (95% CI) =	0.40 (-0.89, 1.69)
P <1,500/Sm negative	94/159	1.00	AP (95% CI) =	N/A
P ≥1,500/Sm negative	47/45	1.62 (0.98, 2.68)	SI (95% CI) =	N/A
P <1,500/Sm positive	30/78	0.71 (0.42, 1.18)	Product term P_1500+_ x Sm:	
P ≥1,500/Sm positive	23/22	1.73 (0.90, 3.34)	OR (95% CI) =	1.51 (0.62, 3.64)
*Plasmodium*-hookworm (n = 601)^c^			RERI (95% CI) =	0.25 (-1.08, 1.58)
P negative/Hk negative	26/46	1.00	AP (95% CI) =	0.17 (-0.69, 1.03)
P positive/Hk negative	146/227	1.01 (0.58, 1.76)	SI (95% CI) =	2.28 (0.01, 607.09)
P negative/Hk positive	9/13	1.18 (0.43, 3.24)	Product term P x Hk:	
P positive/Hk positive	62/72	1.45 (0.78, 2.68)	OR (95% CI) =	1.21 (0.41, 3.57)
*Plasmodium*_1500+_-hookworm (n = 601)^c^			RERI (95% CI) =	0.42 (-1.27, 2.11)
P <1,500/Hk negative	115/213	1.00	AP (95% CI) =	0.18 (-0.45, 0.81)
P ≥1,500/Hk negative	57/60	1.58 (1.01, 2.47)*	SI (95% CI) =	1.46 (0.35, 6.10)
P <1,500/Hk positive	48/68	1.35 (0.86, 2.12)	Product term P_1500+_ x Hk:	
P ≥1,500/Hk positive	13/17	2.35 (1.19, 4.62)*	OR (95% CI) =	1.10 (0.47, 2.62)
**Splenomegaly co-infection categories**	**N enlarged/normal spleen**	**OR (95% CI)**	**Interaction measures**^d^	
*Plasmodium-S*. *haematobium* (n = 435)^a^			RERI (95% CI) =	-0.12 (-2.45, 2.20)
P negative/Sh negative	5/44	1.00	AP (95% CI) =	N/A
P positive/Sh negative	82/206	2.48 (0.89, 6.96)	SI (95% CI) =	N/A
P negative/Sh positive	2/19	0.91 (0.15, 5.65)	Product term P x Sh:	
P positive/Sh positive	17/60	2.27 (0.72, 7.18)	OR (95% CI) =	1.01 (0.15, 6.97)
*Plasmodium*_1500+_*-S*. *haematobium* (n = 435)^a^			RERI (95% CI) =	-0.28 (-1.78, 1.22)
P <1,500/Sh negative	54/193	1.00	AP (95% CI) =	N/A
P ≥1,500/Sh negative	33/57	1.42 (0.80, 2.51)	SI (95% CI) =	N/A
P <1,500/Sh positive	14/64	0.94 (0.46, 1.91)	Product term _P1500+_ x Sh:	
P ≥1,500/Sh positive	5/15	1.08 (0.35, 3.30)	OR (95% CI) =	0.81 (0.21, 3.12)
*Plasmodium*-*S*. *mansoni* (n = 490)^b^			RERI (95% CI) =	-0.53 (-2.83, 1.76)
P negative/Sm negative	5/44	1.00	AP (95% CI) =	N/A
P positive/Sm negative	82/206	2.78 (0.99, 7.78)	SI (95% CI) =	N/A
P negative/Sm positive	1/21	0.62 (0.06, 5.96)	Product term P x Sm:	
P positive/Sm positive	24/107	1.86 (0.62, 5.54)	OR (95% CI) =	1.08 (0.11, 11.01)
*Plasmodium*_1500+_-*S*. *mansoni* (n = 490)^b^			RERI (95% CI) =	-0.36 (-1.47, 0.74)
P <1,500/Sm negative	54/193	1.00	AP (95% CI) =	N/A
P ≥1,500/Sm negative	33/57	1.46 (0.83, 2.57)	SI (95% CI) =	N/A
P <1,500/Sm positive	15/93	0.74 (0.38, 1.46)	Product term P_1500+_ x Sm:	
P ≥1,500/Sm positive	10/35	0.84 (0.37, 1.90)	OR (95% CI) =	0.77 (0.26, 2.32)
*Plasmodium*-hookworm (n = 592)^c^			RERI (95% CI) =	-3.49 (-9.27, 2.28)
P negative/Hk negative	5/67	1.00	AP (95% CI) =	-1.62 (-3.84, 0.60)
P positive/Hk negative	104/263	3.84 (1.43, 10.26)*	SI (95% CI) =	0.25 (0.07, 0.91)*
P negative/Hk positive	3/17	2.81 (0.56, 14.06)	Product term P x Hk:	
P positive/Hk positive	21/112	2.16 (0.74, 6.29)	OR (95% CI) =	0.20 (0.04, 1.09)
*Plasmodium*_1500+_-hookworm (n = 592)^c^			RERI (95% CI) =	-0.28 (-1.29, 0.74)
P <1,500/Hk negative	69/255	1.00	AP (95% CI) =	N/A
P ≥1,500/Hk negative	40/75	1.37 (0.83, 2.26)	SI (95% CI) =	N/A
P <1,500/Hk positive	16/97	0.70 (0.37, 1.33)	Product term P_1500+_ x Hk:	
P ≥1,500/Hk positive	8/32	0.79 (0.34, 1.88)	OR (95% CI) =	0.82 (0.27, 2.50)

^a^*S*. *mansoni-*positive individuals excluded from model; ORs are adjusted for sex, age group, socioeconomic status, soil-transmitted helminth infection, and malnutrition

^b^*S*. *haematobium-*positive individuals excluded from model; ORs are adjusted for sex, age group, socioeconomic status, soil-transmitted helminth infection, and malnutrition

^c^ORs are adjusted for sex, age group, socioeconomic status, *Schistosoma* infection, and malnutrition

^d^Interaction measures on additive scale: relative excess risk due to interaction (RERI), attributable proportion (AP), and synergy index (SI). Interaction measure on multiplicative scale assessed by product term introduced in the multivariable logistic model

*Statistically significant with p <0.05

Parasite species abbreviations: *Plasmodium* (P), high *Plasmodium* parasitemia with ≥1,500 parasites/μl of blood (P_1500+_), *S*. *mansoni* (Sm), *S*. *haematobium* (Sh), and hookworm (Hk).

N/A = not applicable due to OR<1 for one or more co-infection categories

### Interaction measures on splenomegaly

Within the national school-based survey, co-infection with *Plasmodium* and either of the two *Schistosoma* species showed antagonistic effects on splenomegaly (Table 3). For the scenarios of high *Plasmodium* parasitemia and *S*. *haematobium* co-infection (OR 0.33, 95% CI 0.13, 0.86) and *Plasmodium*-*S*. *mansoni* (OR 0.32, 95% CI 0.10, 0.99) co-infection, these negative interactions were found to be on multiplicative level. Both single-infection categories of the two scenarios showed significantly higher ORs for splenomegaly; the co-infection categories, however, were below the expected sum or non-significant. No significant antagonism or synergism on splenomegaly for *Plasmodium*-hookworm co-infection was observed in the school-based sample.

Table 4 summarizes the six scenarios applied for testing interaction on splenomegaly in *Plasmodium*-helminth co-infection in school-aged children/adolescents from the community-based surveys. Neither of the *Plasmodium*-*Schistosoma* pairs showed any significant interaction measures or higher ORs for single and co-infection categories compared to the reference categories for splenomegaly. For *Plasmodium*-hookworm co-infection, however, we could identify a significant antagonism for splenomegaly on additive scale (SI 0.25, 95% CI 0.07, 0.91).

## Discussion

This study presents an analysis of interaction measures on additive and multiplicative scales for *Plasmodium*-helminth co-infection on two clinical manifestations (i.e., anemia and splenomegaly) using a suite of data from cross-sectional surveys carried out in Côte d’Ivoire in two population groups (i.e., school-aged children/adolescents and adults). Our findings revealed only a limited contribution of (co-)infections on anemia in adults. In adult females, pregnancies and motherhood may have much higher impact on anemia, especially in rural settings of Côte d’Ivoire where a woman has, on average, five or six children in her life [42]. Another explanation for the low contribution may be seen in the low helminth infection intensities in most of the positive cases [17]. In a recent study, Chami and colleagues have shown that general infection status contributes little to the anemia burden in adults, however cure and reduction of heavy intensity infections of *S*. *mansoni* and hookworm may reduce anemia in affected adults up to 37.0% and 24.9%, respectively [43]. Yet, in our adult sample *S*. *mansoni*-co-infection showed a significant antagonistic effect on anemia on multiplicative scale; a finding that warrants further investigation. Splenomegaly rates were low in adults, and hence, it was not feasible to pursue any specific analysis. The low rates are likely attributable to an acquired immunity to malaria among this age strata in this high-endemicity area [44]. In the school-aged/adolescent population, we observed considerable differences depending on whether the study was community- or school-based. School-aged children/adolescents from the four communities in southern Côte d’Ivoire showed higher infection prevalence, levels of parasitemia and clinical morbidity than the national school-based sample. Differences in included and excluded *Schistosoma*-infected individuals in the communities can be explained by higher peak age in *S*. *mansoni* infections and a more developed infrastructure in the village of Sahoua where most of the *S*. *haematobium* cases occurred. Relationships between clinical morbidity and single infections with *Schistosoma* and *Plasmodium*, yet, were found to be more significant and pronounced among school children from a more heterogeneous sample from whole Côte d’Ivoire. However, all identified significant interactions in both school-aged children/adolescents data sets are of antagonistic nature but vary in magnitude and parasite species combination. Each *Plasmodium*-helminth pair for the school-aged/adolescents strata is discussed in more detail below.

### *Plasmodium*-hookworm co-infection

Unlike studies conducted in East Africa [45, 46] and what is generally expected from two parasite species that have been shown to be positively associated [47] and having different mechanistic pathways of causing anemia [2, 14], we could not identify any exacerbation in anemia due to *Plasmodium*-hookworm co-infection in the school-aged population. What we observed is basically no departure from additivity. This may partly be explained by generally low hookworm infection intensities found in our study samples [10, 17]. Our results are in line with earlier findings from Côte d’Ivoire [16] and from rural communities in central Ghana [47] that showed a comparable epidemiologic profile with high *Plasmodium* rates (≥75% of school-aged children/adolescents infected) and mainly low-intensity hookworm infections. In settings with such a high force of *Plasmodium* transmission, contribution to anemia may thus be largely attributable to malaria, while the effect on anemia from light-intensity hookworm infection seems negligible. Nutritional studies assessing potential contribution of *Plasmodium*-hookworm co-infection to iron-deficiency anemia undertaken in rural Côte d’Ivoire further emphasize this assumption [5]. Of note, numerous studies on *Plasmodium*-helminth co-infection and its relationship with anemia reported highest odds of anemia in *Plasmodium* single-infected individuals, followed by helminth (co-)infected individuals compared to the uninfected reference group [48–50]. However, these prior studies did not have a closer look at potential (antagonistic) interactions, which indeed seem to be present, considering lower risks of anemia in people co-infected compared to *Plasmodium* single-infected individuals. Within the community-based survey, *Plasmodium-*hookworm co-infection showed protective effects against splenomegaly, whereas this negative interaction could not be confirmed in the national school-based sample. Other studies on potential interaction from *Plasmodium*-hookworm co-infection on splenomegaly are still scarce. Mboera and colleagues investigated the effect of concurrent hookworm and *Plasmodium* infection in Tanzanian school children and revealed enhanced *Plasmodium* parasitemia in *Plasmodium*-hookworm co-infected individuals but no exacerbating effect on splenomegaly [49].

### *Plasmodium-S*. *haematobium* co-infection

In the community-based sample where *S*. *haematobium* infection was three times more prevalent than in the national school-based sample, *S*. *haematobium* single or *Plasmodium- S*. *haematobium* co-infection were negatively associated with anemia, whereas *Plasmodium* infection was the main driver of splenomegaly. For all four scenarios of *Plasmodium*-*S*. *haematobium* co-infection on anemia and splenomegaly, we found no significant interactions among school-aged children/adolescents from the four communities. Yet, we identified a significant antagonism on multiplicative scale on both morbidity indicators for the scenario of *S*. *haematobium* plus concomitant high *Plasmodium* parasitemia from the national school-based survey. Earlier studies from Tanzania and Zimbabwe identified *Plasmodium* and *S*. *haematobium* single- and co-infections as important predictors of anemia and splenomegaly, while only little information was available on the presence and magnitude of potential interactions from co-infection [20, 49, 50]. Findings from Mali and Senegal corroborate the assumption of a protective effect of *Plasmodium-S*. *haematobium* co-infection on malaria pathology, such as a reduced number of febrile malaria episodes and lower *Plasmodium* parasitemia in co-infected individuals [15, 18, 51, 52].

### *Plasmodium-S*. *mansoni* co-infection

While earlier studies conducted in East Africa reported exacerbation of (hepato-)splenomegaly in *Plasmodium*-*S*. *mansoni* co-infection [8, 19], we observed the contrary. Although all infection categories (single and co-infection) were significantly positively associated with splenomegaly in school-aged children/adolescents from the national school-based sample, interaction measures clearly indicated an antagonism on multiplicative scale in *Plasmodium-S*. *mansoni* co-infected school-aged children/adolescents. Among school-aged children/adolescents from the four communities, we could not identify a significant antagonism, but odds of splenomegaly seemed to be reduced both in school-aged children/adolescents with low and high *Plasmodium* parasitemia and concomitant *S*. *mansoni* infection, compared to school-aged children/adolescents infected with *Plasmodium* singly (low and high parasitemia). The odds of anemia were highest among *Plasmodium*-*S*. *mansoni* co-infected, compared to all other infection categories, in school-aged children/adolescents from the school-based sample. Interaction analysis, however, did not show any protective effect nor any exacerbation to the expected risk from concomitant infection. Interestingly, both antagonism as well as synergism in *Plasmodium-S*. *mansoni* co-infection might be explained by immunological interactions by down- or up-regulating, respectively, of a pro-inflammatory immune response that is related with the development of (hepato-)splenomegaly and that may also contribute to anemia due to inflammation [1, 53, 54]. The direction in which the immune response and thus the potential morbidity is induced may depend on the clinical phase of the disease, the duration since infection, the infection intensity of both, *Plasmodium* and *Schistosoma*, and the disposition of the host’s immune system [53]. In our study, we mainly focused on school-aged children/adolescents, part of whom might not yet have acquired full immunity against these infectious diseases. All *S*. *mansoni*-positive individuals were identified as egg excreters and their infection stage thus may be considered as chronic but with mainly low egg counts.

### Implications for public health

Although we mainly identified antagonistic effects on anemia and splenomegaly in *Plasmodium*-helminth co-infected individuals, our data indicate considerable burden due to these infections. Especially on national scale, multiparasitism represented a double burden among school-aged children/adolescents, yet an exacerbation from concomitant infection was not evident in our setting. Burden assessment due to parasitic infections for co-endemic countries thus warrants adaption as demanded and discussed over the last decade [55, 56]. Of note are also the apparent differences in interactions of *Plasmodium*-helminth co-infections between African sub-regions. While data from East African settings highlight potential exacerbation [19–21] the opposite was found in studies investigating etiopathology of malaria in helminth co-infected individuals in West Africa [15, 16, 18, 51, 52]. Some of these differences from one country to another may be explained through a different force of *Plasmodium* transmission (e.g., mostly higher in West Africa), but also varying helminth species strains, related virulence and, to what extent they cause clinical morbidity, may play a role whether *Plasmodium*-helminth co-infection results in a more antagonistic effect rather than in synergism [9, 57]. Moreover, the presence of a second helminth co-infection may steer potential interactions on malaria pathology in the opposite direction or enhance them [58, 59]. In the light of national control programs and increased financial means and control efforts targeting helminthiases, a deeper understanding about potential effects on susceptibility to malaria in co-endemic areas should be gained. Removing helminth infections may alter immune responses to *Plasmodium* infection in previously helminth-infected individuals and thus also influence the number of malaria attacks and severity [58]. Malaria control should thus pay increased attention to the school-aged population that is the major target group of preventive chemotherapy against helminthiases and already neglected in terms of malaria treatment and use of preventive measures compared to other populations (e.g., pregnant women and children under five years of age). Furthermore, for enhancing (cost-)efficiency, concerted efforts against both types of infectious diseases in co-endemic areas are desirable and could for example include education campaigns against malaria during helminth mass drug administration and against helminthiases during mass distributions of insecticide-treated bed nets, respectively.

### Strengths and limitations

Our study has several strengths and limitations. The diagnostic approach, particularly for detection of helminth infections, lacks sensitivity, and hence infection rates might be underestimated. Of note, mainly light-intensity infections were missed, thus our results might still be reliable for relationship and interaction analysis between infection status and clinical morbidity [7]. In Côte d’Ivoire, helminthiases other than schistosomiasis and soil-transmitted helminthiasis exist, such as enterobiasis, strongyloidiasis, onchocerciasis, and lymphatic filariasis [60, 61]. These diseases were not investigated but they might influence malaria pathology by altering the immune response to *Plasmodium* [62, 63]. With regard to the two clinical outcomes considered in our study—anemia and splenomegaly—especially the former is multifactorial and may be caused by hematological diseases, inflammation, and nutritional deficiencies, in addition to helminth infection and malaria. However, we conjecture that a major contribution to these clinical manifestations are parasitic infections [1] with *Plasmodium* and helminth infections ranking among the most important ones in terms of prevalence and burden [55]. With regard to methodological and analytical limitations it is worth highlighting that especially the concept of biologic interaction on additive scale, as proposed by Rothman et al. [22], is designed for case-control studies with matched groups and investigating risk exposures (relative risk (RR) or OR >1); conditions that are usually not met in observational studies. Cross-sectional studies may thus complicate analysis or produce a high uncertainty and variance in the interaction estimates through (i) unbalanced numbers within the different exposure categories (e.g., low number of *Plasmodium*-uninfected school-aged children/adolescents in our samples); (ii) relationships between exposure and outcome may not always reveal an actual risk but rather being protective (RR or OR <1) compared to the defined reference category; (iii) the investigated outcomes may be influenced by other covariates (e.g., age, sex, and socioeconomic status) that are potentially not equally distributed among the comparison groups; and (iv) splenomegaly and anemia are manifestations due to chronic infection and longitudinal study designs may better capture relationships with (earlier) infections that may even be missed during cross-sectional surveys. We addressed some of these points by the introduction of low- and high-parasitemia *Plasmodium* infection scenarios to avoid small reference groups and for investigation of the relationship with parasite burden. Additionally, effects on estimates from unbalanced numbers in each category may be less pronounced in large data sets such as the national school-based study sample in the current analysis. Interaction measures on additive scale for preventive factors were not considered and recoding of exposure variables, as proposed by Knol and colleagues [41], avoided due to complication of subsequent interpretation. Furthermore, we built logistic regression models that allowed for adjustment for potential confounders and *Schistosoma* species-specific models by excluding individuals with concomitant infections with the second *Schistosoma* species. To reassure that only individuals from actual co-endemic areas are compared within interaction analysis, we subsampled school-aged children/adolescents from the national cohort according to helminth endemicity. This may have helped to harmonize partly as seen in several differences in parameters between excluded and included individuals. We could, however, not change the study design; a longitudinal design looking at infection status at different time points and thus allowing for discrimination of repeated infections may be more appropriate in future studies assessing clinical outcomes that are related to chronic exposure with the investigated parasites.

## Supporting information

S1 Table Interaction measures on anemia for *Plasmodium*-helminth co-infection among adults (≥19 years) from four community-based surveys conducted between 2011 and 2013 in southern Côte d’Ivoire. (DOCX)

S1 Fig Prevalence of anemia by *Plasmodium* parasitemia category for school-aged children/adolescents from four community-based and a national school-based study conducted between 2011 and 2013 in Côte d’Ivoire. A: in all school-aged children/adolescents from the community-based studies; B: in school-aged children/adolescents from the community-based studies stratified by sex; C: in all school-aged children/adolescents from the national school-based study; D: in school-aged children/adolescents from the school-based study stratified by sex.
(TIF)

S1 Data Data used for analysis of community cohorts (school-aged children/adolescents and adults). (XLSX)

S2 Data Data used for analysis of school-based cohort. (XLSX)
