# Supplementary material for: Antagonistic effects of Plasmodium-helminth co-infections on malaria pathology in different population groups in Côte d’Ivoire
Source: PLoS Negl Trop Dis. 2019 Jan 10;13(1):e0007086. doi: 10.1371/journal.pntd.0007086 (PMC6343929; doi:10.1371/journal.pntd.0007086)
Supplement: S1 Table — (DOCX) [file pntd.0007086.s001.docx]

**Table S1. Interaction measures on anemia for *Plasmodium*-helminth co-infection among adults (≥19 years) from a community-based survey.**

| **Co-infection categories** | **N anaemic/non-anaemic** | **OR (95% CI)** | **Interaction measures^d^** | | |
| --- | --- | --- | --- | --- | --- |
| *Plasmodium - S. haematobium* (n=559)^a^ | |  | RERI (95% CI) = | | -0.18 (-1.53, 1.17) |
| P negative/Sh negative | 84/209 | 1.0 | AP (95% CI) = | | N/A |
| P positive/Sh negative | 76/159 | 1.23 (0.84, 1.80) | SI (95% CI) = | | N/A |
| P negative/Sh positive | 4/14 | 0.71 (0.22, 2.24) | Product term P x Sh: | | |
| P positive/Sh positive | 3/10 | 0.76 (0.20, 2.89) | OR (95% CI) = | | 0.88 (0.15, 5.09) |
|  |  |  |  | |  |
| *Plasmodium* - *S. mansoni* (n=672)^b^ | |  | RERI (95% CI) = | | -0.81 (-1.69, 0.08) |
| P negative/Sm negative | 84/209 | 1.0 | AP (95% CI) = | | N/A |
| P positive/Sm negative | 76/159 | 1.22 (0.84, 1.79) | SI (95% CI) = | | N/A |
| P negative/Sm positive | 20/51 | 1.11 (0.61, 1.99) | Product term P x Sm: | | |
| P positive/Sm positive | 12/61 | 0.52 (0.26, 1.03) | OR (95% CI) = | | **0.39 (0.16, 0.95)*** |
|  |  |  |  | |  |
| *Plasmodium* - Hookworm (n=706)^c^ | |  | RERI (95% CI) = | | -0.27 (-1.06, 0.52) |
| P negative/Hk negative | 76/195 | 1.0 | AP (95% CI) = | | N/A |
| P positive/Hk negative | 63/150 | 1.09 (0.73, 1.64) | SI (95% CI) = | | N/A |
| P negative/Hk positive | 33/80 | 1.15 (0.70, 1.89) | Product term P x Hk: | | |
| P positive/Hk positive | 28/81 | 0.97 (0.58, 1.63) | OR (95% CI) = | 0.77 (0.38, 1.59) | |

^a^*S. mansoni* positive individuals excluded from model; ORs are adjusted for sex, age group, socioeconomic status, STH infection, and malnutrition.

^b^*S. haematobium* positive individuals excluded from model; ORs are adjusted for sex, age group, socioeconomic status, STH infection, and malnutrition.

^c^ORs are adjusted for sex, age group, socioeconomic status, schistosomiasis infection, and malnutrition.

^d^Interaction measures on additive scale: Relative Excess Risk due to Interaction (RERI), Attributable Proportion (AP), and Synergy index (SI). Interaction measure on multiplicative scale assessed by product term introduced in the multivariable logistic model.

*Statistically significant with p<0.05.

Parasite species abbreviations: *Plasmodium* (P), *S. mansoni* (Sm), *S. haematobium* (Sh), and Hookworm (Hk).

N/A=not applicable due to OR<1 for one or more co-infection categories
