# Supplementary figures and images for: Antagonistic effects of Plasmodium-helminth co-infections on malaria pathology in different population groups in Côte d’Ivoire
Source: PLoS Negl Trop Dis. 2019 Jan 10;13(1):e0007086. doi: 10.1371/journal.pntd.0007086 (PMC6343929; doi:10.1371/journal.pntd.0007086)

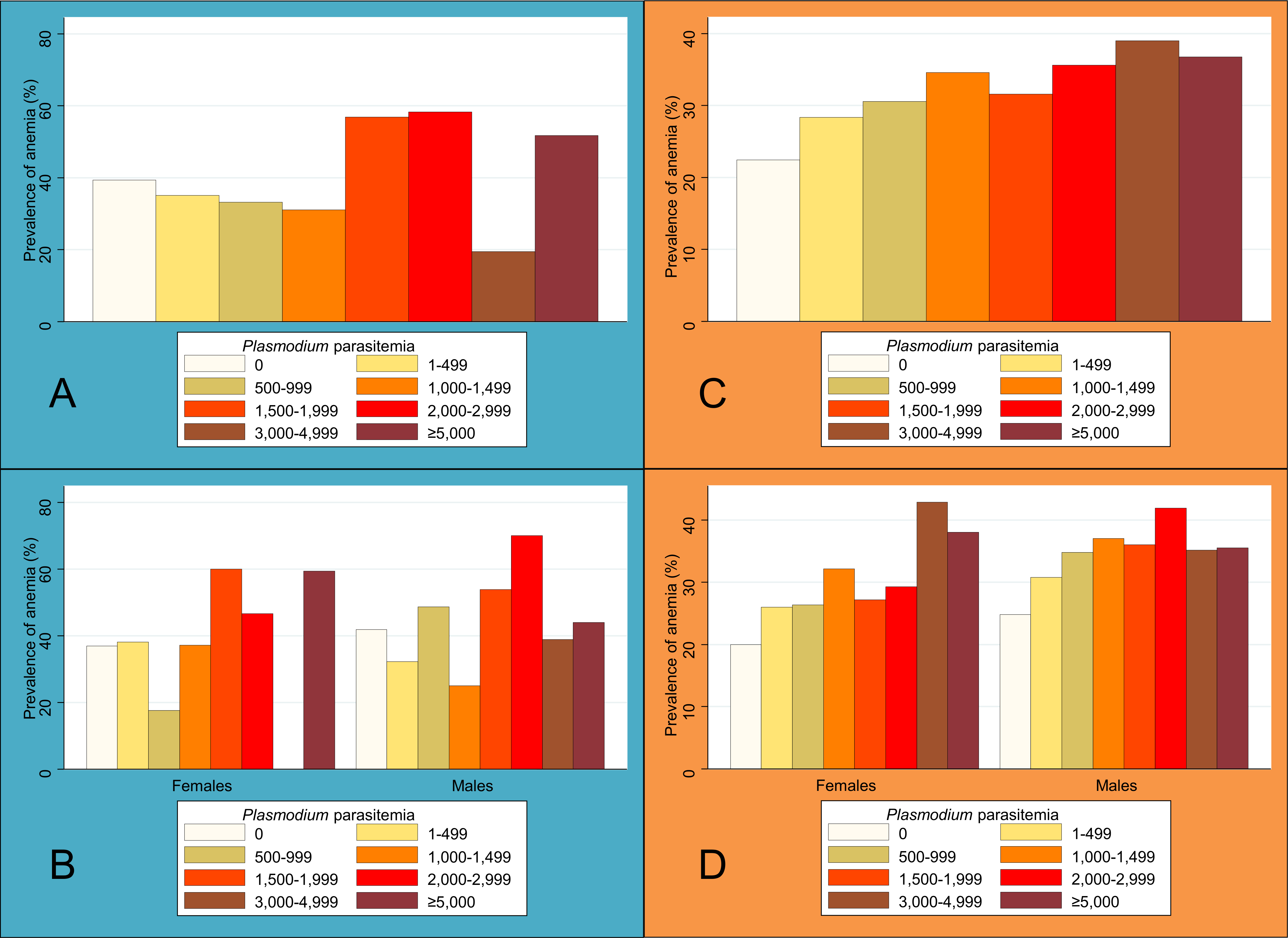

Supplement: S1 Fig — A: in all school-aged children/adolescents from the community-based studies; B: in school-aged children/adolescents from the community-based studies stratified by sex; C: in all school-aged children/adolescents from the national school-based study; D: in school-aged children/adolescents from the school-based study stratified by sex. (TIF) [file pntd.0007086.s002.tif]
